# Establishing Reference Values for Isometric Knee Extension and Flexion Strength

**DOI:** 10.3389/fphys.2021.767941

**Published:** 2021-10-15

**Authors:** Nejc Šarabon, Žiga Kozinc, Mihael Perman

**Affiliations:** ^1^Faculty of Health Sciences, University of Primorska, Izola, Slovenia; ^2^Andrej Marušič Institute, University of Primorska, Koper, Slovenia; ^3^Human Health Department, InnoRenew CoE, Izola, Slovenia; ^4^Laboratory for Motor Control and Motor Behavior, S2P, Science to Practice, Ltd., Ljubljana, Slovenia; ^5^Faculty of Mathematics, Natural Sciences and Information Technologies, University of Primorska, Koper, Slovenia; ^6^Faculty of Mathematics and Physics, University of Ljubljana, Ljubljana, Slovenia

**Keywords:** knee strength, lower limb, muscle capacity, normative, reference values

## Abstract

Single-joint isometric and isokinetic knee strength assessment plays an important role in strength and conditioning, physical therapy, and rehabilitation. The literature, however, lacks absolute reference values. We systematically reviewed the available studies that assessed isometric knee strength. Two scientific databases (PubMed and PEDro) were searched for the papers that are published from the inception of the field to the end of 2019. We included studies that involved participants of both genders and different age groups, regardless of the study design, that involved isometric knee extension and/or flexion measurement. The extracted data were converted to body-mass-normalized values. Moreover, the data were grouped according to the knee angle condition (extended, mid-range, and flexed). A meta-analysis was performed on 13,893 participants from 411 studies. In adult healthy males, the pooled 95% confidence intervals (CI) for knee extension were 1.34–2.23Nm/kg for extended knee angle, 2.92–3.45Nm/kg for mid-range knee angle, and 2.50–3.06Nm/kg for flexed knee angle, while the CIs for flexion were 0.85–1.20, 1.15–1.62, and 0.96–1.54Nm/kg, respectively. Adult females consistently showed lower strength than adult male subgroups (e.g., the CIs for knee extension were 1.01–1.50, 2.08–2.74, and 2.04–2.71Nm/kg for extended, mid-range, and flexed knee angle condition). Older adults consistently showed lower values than adults (e.g., pooled CIs for mid-range knee angle were 1.74–2.16Nm/kg (male) and 1.40–1.64Nm/kg (female) for extension, and 0.69–0.89Nm/kg (male) and 0.46–0.81Nm/kg (female) for flexion). Reliable normative for athletes could not be calculated due to limited number of studies for individual sports.

## Introduction

Assessment of human maximal muscular strength and power ability is routinely performed within strength and conditioning (McMaster et al., 2014; Paul and Nassis, 2015; Suchomel et al., 2016), as well as physical therapy and rehabilitation practice (Dickoff-Hoffman and Davies, 1993; Myer et al., 2006). Strength and power assessment may focus on the capacity of individual muscle groups or the whole kinetic chain (Schrama et al., 2014; Suchomel et al., 2016; Zapparoli and Riberto, 2017; Petrigna et al., 2019). For instance, during the rehabilitation of the anterior cruciate ligament injury, it is common to perform both single-joint knee strength assessment and jump or hop tests for height or distance (a multi-joint task; O’Malley et al., 2018). The body of knowledge on strength and power assessments is continuously growing, with novel measurement devices (Hickey et al., 2018; Whinton et al., 2018; Palmer et al., 2020) and methodological approaches to assessment (Bellumori et al., 2011; García-Ramos et al., 2019; Hughes et al., 2019) being proposed and validated. In terms of single-joint strength assessments, most of the research has been dedicated to the knee joint, which is likely due to the high reliability of these measurements and their implications in rehabilitation (de Araujo Ribeiro Alvares et al., 2015; Muñoz-Bermejo et al., 2019) and implications in the rehabilitation of several prevalent and detrimental lower-limb injuries (Myer et al., 2006; Cvjetkovic et al., 2015; Kaeding et al., 2017; Bourne et al., 2018; Goff et al., 2018; O’Malley et al., 2018). Knee extension and flexion strength measurements have been consistently shown as reliable when conducted under isometric (De Carvalho Froufe Andrade et al., 2013; de Araujo Ribeiro Alvares et al., 2015; Sung et al., 2019) or isokinetic (Sole et al., 2007; De Carvalho Froufe Andrade et al., 2013; de Araujo Ribeiro Alvares et al., 2015; Törpel et al., 2017; Zapparoli and Riberto, 2017; Muñoz-Bermejo et al., 2019) conditions using state-of-the-art dynamometers. Moreover, moderate to high reliability and validity have been reported for knee strength assessments performed by hand-held dynamometers (Mentiplay et al., 2015; Chamorro et al., 2017; Chopp-Hurley et al., 2019; Lesnak et al., 2019; van der Made et al., 2019).

A large body of evidence related to knee strength has facilitated the formulation of guidelines for coaches, physical therapists, and other practitioners within sport science and medicine, orthopedics, rehabilitation, and prevention. For instance, the ratio between hamstring and quadriceps muscles (assessed by the knee flexion and extension torque capacity, respectively) has been shown as one of the risk factors for future lower limb injuries (Devan et al., 2004; Dallinga et al., 2012; Lee et al., 2018) and has been suggested as one of the criteria for safe return to sport (Cvjetkovic et al., 2015; Erickson and Sherry, 2017). Moreover, inter-limb knee strength asymmetries have been linked to history of injury (Schiltz et al., 2009) and reduced sport performance (Bishop et al., 2018). One of the paramount goals of rehabilitation of unilateral knee injuries is to establish the level of strength similar to the uninjured side (Myer et al., 2006; O’Malley et al., 2018). Although some ambiguities exist (Kaplan and Witvrouw, 2019), the guidelines related to inter-limb asymmetries in knee strength as well as flexor to extensor imbalances are useful in prevention and rehabilitation of knee injuries. However, less is known regarding the absolute reference values for knee flexion and extension strength that could be used as a reference in rehabilitation of athletic injuries, prevention or assessing the general functional capacity in older adults or patient populations. In addition to quantifying the ratios between the limbs or opposite muscle groups, it could be useful to have a reference value to which absolute strength levels of an individual could be compared.

Several previous studies have attempted to establish reference values for knee strength in different populations. These studies have encompassed athletes (Zvijac et al., 2014; Risberg et al., 2018; Hannon et al., 2019), children (Holm et al., 2008; McKay et al., 2017), older adults (Pereira et al., 2019), and general adult population (Meldrum et al., 2007; Harbo et al., 2012; Bohannon, 2017; McKay et al., 2017). Therefore, albeit limited, reference values in the literature do exist. However, comparing these studies is difficult, because they reported different units of measurement. Most notably, some studies reported their results as absolute force (Meldrum et al., 2007) or absolute torque values (Harbo et al., 2012; McKay et al., 2017), while others also reported body-mass normalized results (Holm et al., 2008; Zvijac et al., 2014; Risberg et al., 2018; Pereira et al., 2019). Moreover, although the single-joint strength assessments are generally valid and reliable, some of the differences could occur due to measurement errors, use of different devices and different assessment protocols. Finally, several factors pertaining to measurement protocol, such as knee angle during the isometric measurements (Marginson and Eston, 2001; Krishnan and Williams, 2014) or velocity during isokinetic measurements (Perrine and Edgerton, 1978; Grbic et al., 2017), are important to consider in strength assessment. Since these factors are not standardized across studies, the generalization of findings from single studies and translation into practice is further limited. On the other hand, there is a large body of methodological, observational and interventional studies that examined and reported knee strength as one of the outcomes. Examining this body of literature would provide a scoping overview of reference values that could be useful for practitioners during the assessment of their clients’ level of strength, as well as scientists to compare the outcomes from their measurements to typical values obtained across previous investigations. To the best of our knowledge, no attempt has been made to date to review all studies that reported knee flexion or extension strength as an outcome.

The aim of this paper was to review all available studies that reported isometric knee extension and/or flexion strength as assessed during maximal voluntary contraction. Because the body of literature investigating knee strength is very large, we decided to limit our review to isometric contractions in healthy populations, including children, adults, older adults, and both genders. While isokinetic strength is more functionally relevant in some aspects than isometric strength, this decision was based on the fact that most isokinetic dynamometers allow the measurement of isometric strength but not vice versa. Moreover, acceptable to high reliability of hand-held dynamometry to assess isometric knee strength has been reported (Mentiplay et al., 2015; Chamorro et al., 2017; Chopp-Hurley et al., 2019; Lesnak et al., 2019; van der Made et al., 2019). To facilitate the comparison of the studies and study subgroups, we aimed to obtain body-mass normalized torque values. For this purpose, we converted absolute force/torque values into body-mass normalized scores by using appropriate estimates (see section Estimating Body Mass Normalized Torque From Absolute Values for details).

## Materials and Methods

### Search Strategy

The review has not been registered *a priori* in any of the available registers for systematic reviews. PRISMA guidelines were generally followed (Moher et al., 2009), with few exception being made on certain points due to the specific nature of this review. Two scientific databases (PubMed and PEDro) were searched for peer-reviewed English language papers, published from the inception of the field to December 31, 2019 (the search process was initiated in December 2019 and concluded in January 2020). The PubMed database was searched with the following key word combination: (*knee OR lower limb OR leg*) *AND* (*dynamometer OR dynamometry OR hand-held dynamometer OR hand-held OR isometric*) *AND* (*maximal voluntary contraction OR maximal strength OR maximal force OR maximal torque OR peak torque OR peak force OR Fmax*). In the PEDro database, we used single key word “knee strength.” We also reviewed the reference lists of systematic reviews, published since 2010, which we identified during the search process. Microsoft Excel 2016 (Microsoft, Redmond, WA, United States) sheets were used for the article search. First, all articles that seem suitable for inclusion as per title were entered into an Excel sheet. Duplicates were removed manually. In the next stage, abstracts were examined and the articles were either excluded or kept for full-text examination and subsequent data extraction. When a definitive decision could not be made at any stage, the paper was kept for the next stage. The selection process was done independently by two authors, and any dispute was resolved with additional discussion and consulting the third author.

### Eligibility Criteria

The *a priori* eligibility criteria are outlined below in the form of PICOS search tool (Methley et al., 2014):

- Population (P): The only inclusion criterion was that the participants were healthy and without musculoskeletal injuries. We included studies that involved participants of both genders and different age groups. Children (<12years), adolescents (12–18years), adults (18–65years of age), and older adults (<65years of age) were considered. Athletic populations were also considered, but based on *post hoc* decision (due to limited number of studies and high between-study heterogeneity), between-sport comparisons could not be reliably assessed. If a study investigated patient populations, we considered the data from the healthy control group when available.
- Intervention (I): No interventions were considered in this study. In case of interventional studies, the baseline control group values were considered.
- Comparisons (C): Not applicable.
- Outcomes (O): Isometric knee strength, measured as force (N or N/kg) or torque (Nm or N/kg) during maximal voluntary contraction. For the analyses, all results were converted into body mass normalized torque (see section Estimating Body Mass Normalized Torque From Absolute Values for details). The data were accepted if it was obtained by commercial or custom-made isometric dynamometers, or isokinetic dynamometers that enabled isometric measurements, as well as if it was obtained by hand-held dynamometry. If multiple methods were used in a study, we considered the results obtained by the method that we judged to be the more reliable and valid (e.g., isometric or isokinetic dynamometers were chosen over hand-held dynamometry).
- Study design (S): All study designs were accepted, with the exception of case studies and studies that re-analyzed the data from previous publications. For reliability and validity studies, we used the averaged data from multiple trials when available, and median value when the results were reported for each trial separately. Articles that were not peer-reviewed were excluded. Conference abstracts, books, editorials, and response letters were also excluded from the search.

### Data Extraction

Following the inclusion criteria, the extracted data included: (a) means and standard deviations for all eligible data on knee flexion and extension strength; (b) participant data (gender, age, body height, body mass, body mass index, health status, and athletic discipline); (c) measurement characteristics [knee angle, hip angle, repetitions, duration of breaks, duration of sustained contraction, type of dynamometer, and task (unilateral or bilateral)]. The data were carefully entered into Microsoft Excel 2016 (Microsoft, Redmond, WA, United States). Preferably, the data were obtained from tables. If the data were presented only in a graphical form, we used the Adobe Illustrator Software (version CS5, Adobe Inc., San Jose, CA, United States) to accurately determine the means and standard deviations. In case of missing data, the corresponding author of the target article was contacted by e-mail. If no response was received after 21days, the author was contacted again. If the author did not reply to the second inquiry, the data were considered irretrievable. For the data extraction process, the papers were split equally between the three authors. During intimal stages of the data extraction, the authors consulted regularly to keep the system of data extraction coherent. During the process, the authors marked the papers for which they had any difficulties or doubts with data extraction. For these papers, all three authors consulted and reached a consensus.

### Estimating Body Mass Normalized Torque From Absolute Values

The mean normalized values were estimated with the following equation, derived from Taylor’s expansion:

$$\overset{-}{z}\approx \frac{\overset{-}{y}}{\overset{-}{x}}+\frac{2\overset{-}{y}}{{\overset{-}{x}}^{3}}\;s_{x}^{2}-\frac{2}{{\overset{-}{x}}^{2}}\;\rho \;s_{x}s_{y}$$

where z̄, ȳ, and x̄ represent the mean normalized value, mean absolute value and body mass, respectively, while s_y_ and s_x_ are standard deviations of the raw values and body mass. Finally, *ρ* represents a correlation coefficient between strength in absolute values and body mass. This value was held fixed at 0.6 for all of our analyses. Small deviations (±0.05) from this value resulted in trivial changes in final estimates. The value was chosen based on the correlational analysis of several of our databases (850 total participants) containing isometric knee extension and flexion strength assessment. In various subgroups, the correlation of knee extension and flexion strength was moderately correlated with body mass (coefficient range: 0.56–0.62). Standard deviations of the normalized values were further estimated with the following equation which was also based on the Taylor’s expansion:

$$s_{z}^{2}=\frac{{\overset{-}{y}}^{2}}{{\overset{-}{x}}^{4}}\;s_{x}^{2}+\frac{1}{{\overset{-}{x}}^{2}}\;s_{y}^{2}-\frac{2\overset{-}{y}}{{\overset{-}{x}}^{3}}\;\rho \;s_{x}$$

### Data Grouping, Elimination, and Analysis

After the data was extracted and converted to body-mass normalized units (Nm/kg), further decisions were made on how to group the data. First, we decided to eliminate bilateral measurements, as they represented a very small portion of the collected data (<2% of studies). We decided not to calculate the unilateral strength because it is not clear to what extent the bilateral deficit could influence the result. Namely, a recent review showed the bilateral deficit for knee extension strength has ranged from −3.5 to −24.6% across studies (Škarabot et al., 2016). The hip angle during the measurements was almost exclusively (93.6% of studies) set at 90°, with all studies setting it between 75 and 110°. Therefore, we performed no subgrouping based on the hip angle. When multiple hip angles were used, the measurement with an angle closest to 90° was considered. The studies varied more substantially in terms of the knee angle used during the measurements, with some angles being rarely examined. Therefore, we decided to group the data according to the knee angle into three categories: extended knee angle (10–45° of knee flexion), mid-range knee angle (50–70° of knee flexion), and flexed knee angle (80–110°). The preliminary analysis is reported in Supplementary File 1, which provides the data separately for each unique angle, regardless of the small number of studies examining certain angles. In the paper, we focus on the data grouped into the aforementioned three categories based on the knee angle. Due to the small number of studies conducted on athletes, we merged recreational and professional athletes into one group for the main analysis. Namely, the preliminary analysis (Supplementary File 1) showed very similar values for the two groups and no systematic trend for one group to exhibit higher strength.

The data were pooled in Comprehensive Meta Analysis (Version 3, Biostat Inc., Englewood, United States). A random-effects model was applied to calculate pooled mean with respective 95% confidence intervals from means, standard deviations, and sample sizes of individuals studies. The data were analyzed for each age group, gender, and muscle group within each knee angle range.

## Results

### Summary of Search Results and Study Characteristics

**Figure 1:**
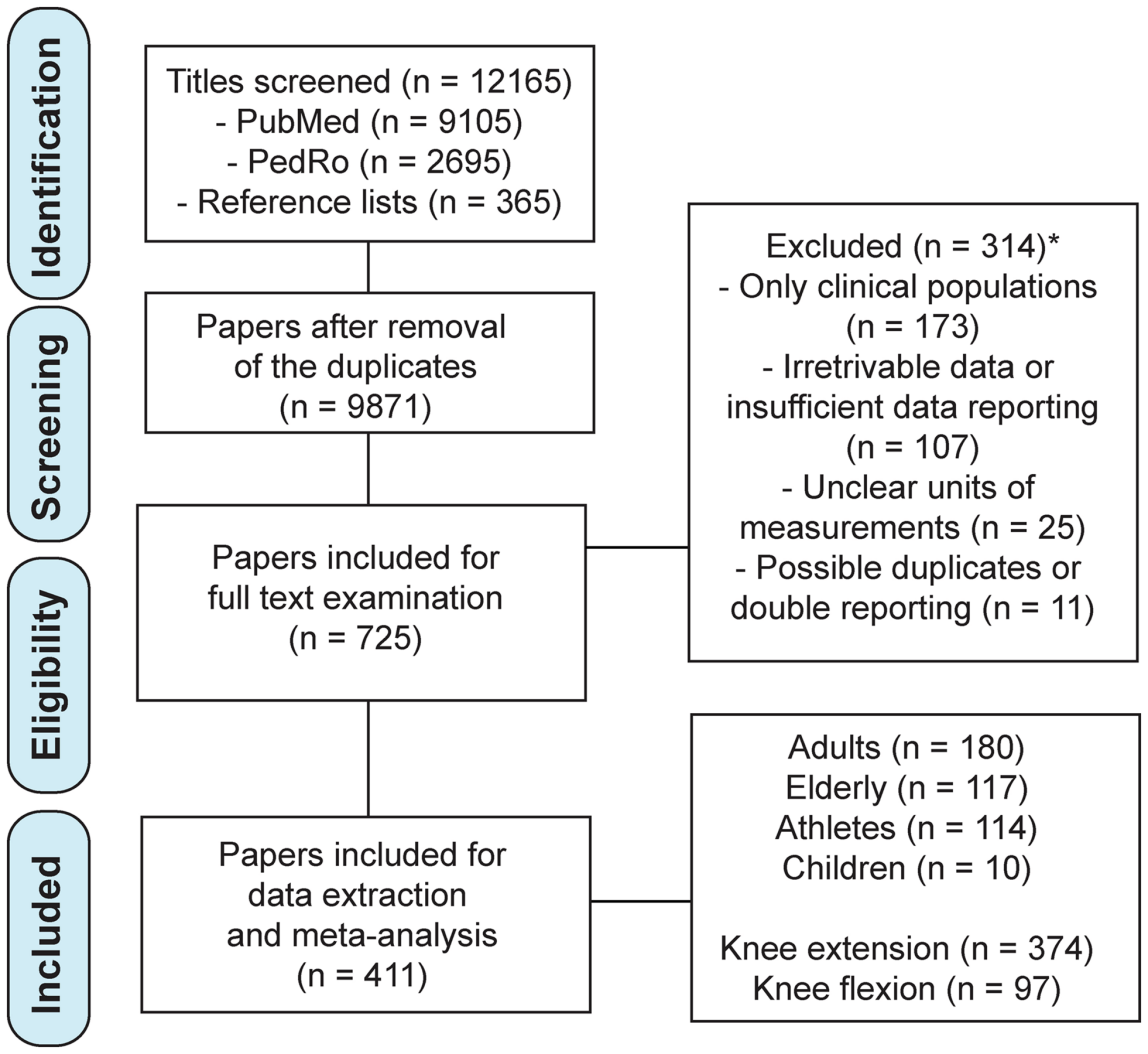
Flowchart of the study selection process. ^*^Note that the numbers of the excluded papers by each reason do not add up to the total, as two papers were excluded for more than one reason.

In total, 12,165 titles were screened (9,105 from the PubMed database, 2,695 from the PEDro database, and 365 additionally identified through reference list searches). In total, 725 articles were chosen for full-text examination. Papers were further excluded due to examining only clinical populations (*n*=173), insufficient or irretrievable data (*n*=107), unclear units of measurements (*n*=25) and for reporting potentially duplicate or overlapping results with other studies (*n*=9). The selection process is also summarized in Figure 1.

Ultimately, we examined 411 studies including 13,893 participants (8,788 non-athlete adult participants in 180 studies, 3,279 elderly participants in 117 studies, 1826 athletes in 114 studies, and 359 children participants in 12 studies). Knee extension strength was reported in 374 studies and knee flexion in 97 studies. These studies were included in the preliminary analysis, which involved no knee angle grouping and considered the data for both genders together and separately. This analysis is available in Supplementary File 1. For the main analyses, we excluded the studies that did not report the values separately for each gender, leaving a total of 12,563 participants in 321 studies (see Table 1 for details).

**Table 1:** Pooled mean strength values with 95% confidence intervals for adult and elderly non-athlete populations.

Groups				Knee strength (Nm/kg)				
Knee angle	Age	Gender	Movement	Mean	95% CI		Participants	Studies
Extended	Adult	F	Extension	1.26	1.01	1.50	95	5
Extended	Adult	F	Flexion	1.00	0.78	1.21	82	5
Extended	Adult	M	Extension	1.79	1.34	2.23	286	10
Extended	Adult	M	Flexion	1.03	0.85	1.20	99	5
Extended	Elderly	F	Extension	0.62	0.18	1.07	40	3
Extended	Elderly	F	Flexion	0.58	0.06	1.10	60	5
Extended	Elderly	M	Extension	1.16	0.95	1.37	132	9
Extended	Elderly	M	Flexion	0.89	0.59	1.20	42	3
Mid-range	Adult	F	Extension	2.38	2.02	2.74	707	15
Mid-range	Adult	F	Flexion	0.94	0.82	1.06	66	4
Mid-range	Adult	M	Extension	3.19	2.92	3.45	937	15
Mid-range	Adult	M	Flexion	1.39	1.15	1.62	832	7
Mid-range	Elderly	F	Extension	1.52	1.40	1.64	1,156	19
Mid-range	Elderly	F	Flexion	0.64	0.46	0.81	78	6
Mid-range	Elderly	M	Extension	1.95	1.74	2.16	534	14
Mid-range	Elderly	M	Flexion	0.79	0.69	0.89	58	4
Flexed	Adult	F	Extension	2.37	2.04	2.71	621	10
Flexed	Adult	F	Flexion	1.07	0.46	1.69	32	2
Flexed	Adult	M	Extension	2.78	2.50	3.06	3,718	27
Flexed	Adult	M	Flexion	1.25	0.96	1.54	267	12
Flexed	Elderly	F	Extension	1.33	1.05	1.62	214	11
Flexed	Elderly	F	Flexion	0.45	0.34	0.56	60	4
Flexed	Elderly	M	Extension	1.77	1.50	2.04	392	15
Flexed	Elderly	M	Flexion	0.70	0.57	0.83	42	3

M, male; F, female; CI, confidence interval.

The number of repetitions per muscle group in the MVC task was most often set to 3 (237 studies) or 2 (125 studies). A minority of studies used a different number of repetitions (one repetition in 13 studies, four repetitions in 17 studies, five repetitions in 12 studies, six repetitions in six studies, and eight repetitions in one study). The duration of contraction also varied substantially between the studies, with 5s (187 studies) and 3s (90 studies) being most common, while other contraction durations were less frequently incorporated (2–3s in 62 studies, 4s 24 studies, 3–5s in 17 studies, 6s in 15 studies, 2s in eight studies, and 3–4s in eight studies). Most frequently, the breaks between the repetitions were set to 60s (118), 180s (72 studies), 30s (70 studies), or 120s (66 studies). Less often, the breaks were set to 90s (27 studies), 20s (25 studies), 40s (12 studies), 15s (eight studies), 10s (eight studies), 300s (four studies), and 240s (one study).

### Knee Strength in Non-athlete Populations

**Figure 2:**
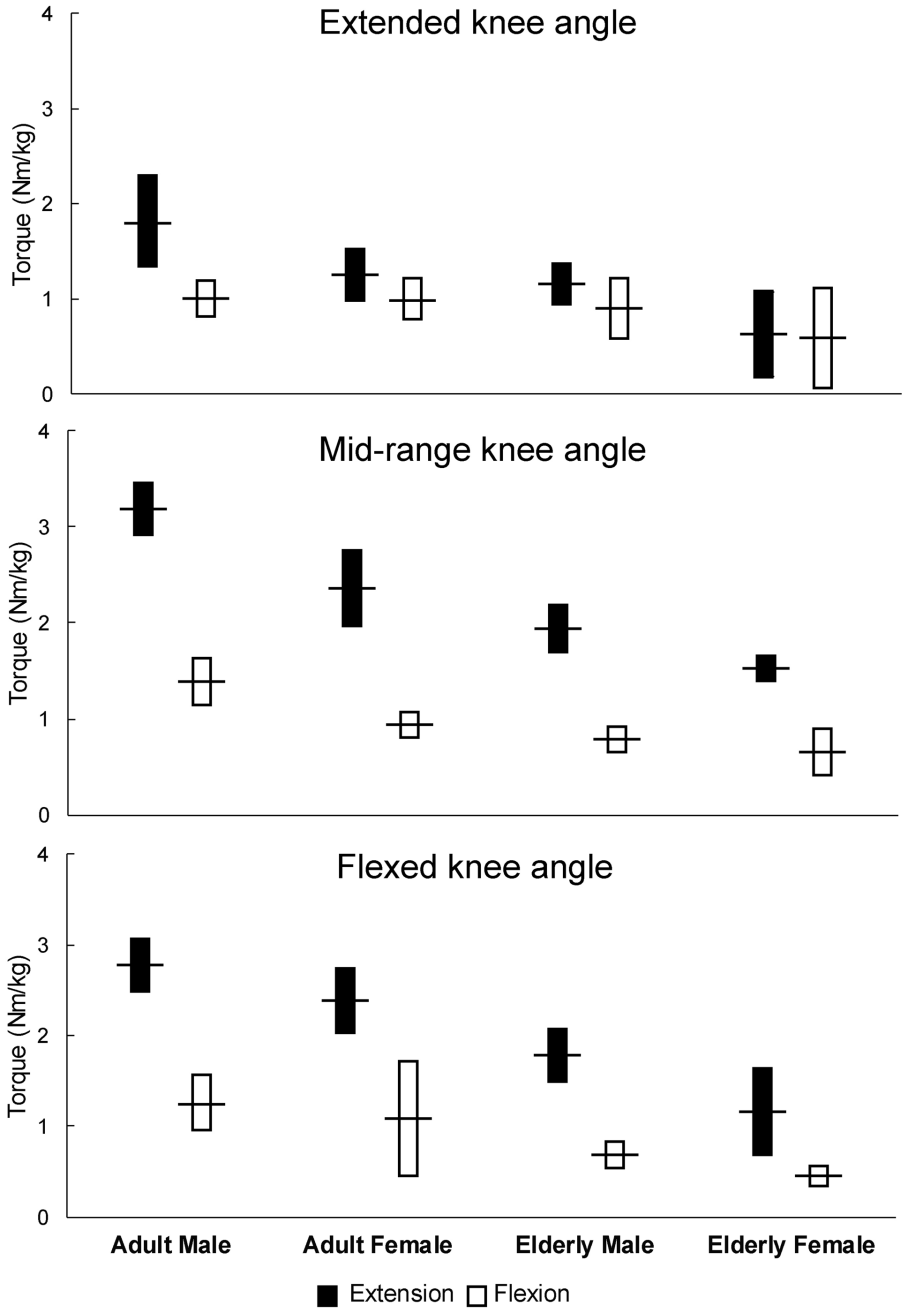
Pooled mean strength values with 95% confidence intervals for adult and elderly non-athlete populations (separated by knee angle).

**Figure 3:**
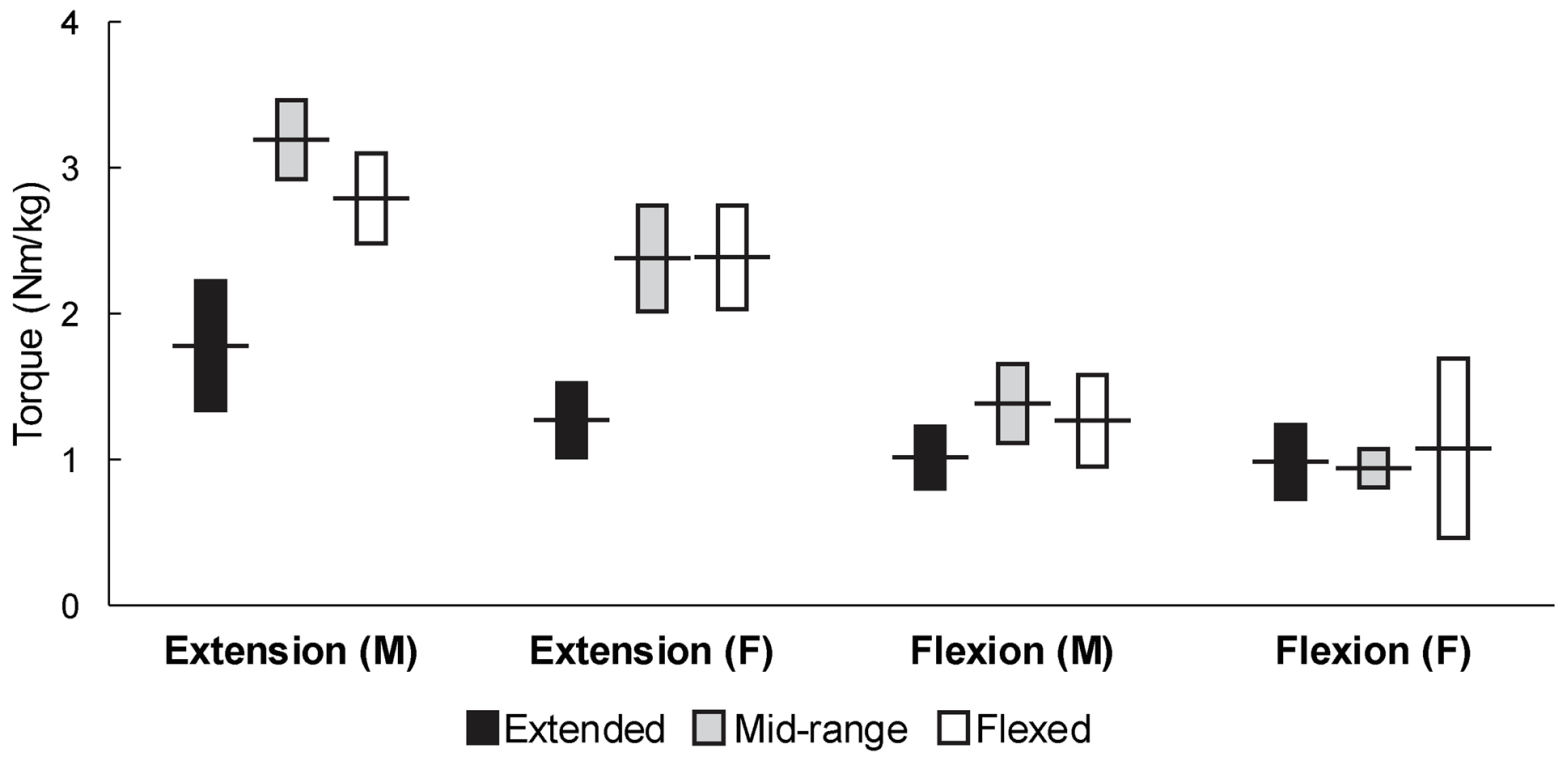
Pooled mean strength values with 95% confidence intervals for adult non-athlete populations across knee angle conditions.

Figure 2 depicts pooled mean strength values with 95% confidence intervals for adult and elderly non-athlete populations, separated by knee angle. In Figure 3, the data for adult population are also shown with different knee angle conditions merged. Within these analyses (i.e., adult and elderly non-athlete participants), 10,550 participants from 213 studies were included. The detailed numbers of studies and participants per group are shown in Table 1. Some groups were poorly represented. Most notably, only two studies (32 participants) included knee flexion strength at flexed knee angle in adult females. Note that older adults were considered as one group because the mean age across studies was very homogenous (i.e., 65–75 in >90% of the studies). For each of the analyses, we also calculated the correlation between mean strength and mean age, which indicated no relationships between the two variables (*r*=−0.12 to 0.04 for extension; *r*=−0.22 to 0.09 for flexion).

When the knee angle was in the extended position during the measurement (Figure 2, top chart), the knee extension and knee flexion mean torque values were most similar, though there were substantial differences in adult males, for which the pooled torque was 1.79Nm/kg (1.34–2.23) for the knee extension and 1.03Nm/kg (0.85–1.20) for the knee flexion. In adult females, the pooled torque was 1.26Nm/kg (1.01–1.50) for knee extension and 1.00Nm/kg (0.78–1.21) for the knee flexion. In elderly males, the pooled torque was 1.16Nm/kg (0.95–1.37) for the knee extension and 0.89Nm/kg (0.85–1.20) for the knee flexion. In elderly females, the pooled torque was 0.62Nm/kg (0.18–1.07) for the knee extension and 0.58Nm/kg (0.06–1.10) for the knee flexion.

When the knee angle was in the mid-range position during the testing, the pooled torque was 3.19Nm/kg (2.92–3.45) for the knee extension and 1.39Nm/kg (1.15–1.62) for the knee flexion. In adult females, the pooled torque was 2.38Nm/kg (2.02–2.74) for knee extension and 0.94Nm/kg (0.82–1.06) for the knee flexion. In elderly males, the pooled torque was 1.95Nm/kg (1.74–2.16) for the knee extension and 0.79Nm/kg (0.69–0.89) for the knee flexion. In elderly females, the pooled torque was 1.52Nm/kg (1.40–1.64) for the knee extension and 0.64Nm/kg (0.46–0.81) for the knee flexion.

Finally, when the knee angle was in the flexed position during the testing (Figure 2, bottom chart), the pooled torque was 2.78Nm/kg (2.50–3.06) for the knee extension and 1.25Nm/kg (0.96–1.54) for the knee flexion. In adult females, the pooled torque was 2.37Nm/kg (2.04–2.71) for the knee extension and 1.07Nm/kg (0.46–1.69) for the knee flexion. In elderly males, the pooled torque was 1.77Nm/kg (1.50–2.04) for the knee extension and 0.70Nm/kg (0.57–0.83) for the knee flexion. In elderly females, the pooled torque was 1.33Nm/kg (1.05–1.62) for the knee extension and 0.45Nm/kg (0.34–0.56) for the knee flexion.

Very few studies were done on children and adolescents. For the former, the data were limited computed to the 90° knee angle only. The knee extension pooled torque was 1.86Nm/kg (1.45–2.27) and 1.37Nm/kg (1.02–1.73) for male and female children, respectively. The knee flexion pooled torque was 1.12Nm/kg (0.78–1.45) and 1.09Nm/kg (0.87–1.31) for male and female children, respectively.

### Knee Strength in Athlete Populations

Substantially fewer studies were conducted on athletes, which precluded between-sport comparisons. Therefore, only “overall” normative for athletes were calculated. It has to be noted that these values consequently present only a summary of the literature and practitioners are encouraged to seek sport-specific data in individual studies. Moreover, in the extended knee angle condition, only the data for male athletes could be pooled. In the flexed knee angle condition, it was possible to pool knee extension strength data, but not knee flexion data. Across all knee angles, 1,779 male athletes from 97 studies were analyzed. The mid-range knee angle condition analysis included 702 participants from 49 studies (560 male athletes from 41 studies and 142 female athletes from eight studies). The detailed results are available in Supplementary File 1.

## Discussion

This paper aimed to obtain reference values for knee strength in athletes and general adult and older adult populations. For this purpose, we performed a broad review of studies that assessed and reported isometric knee extension and/or flexion strength during maximal voluntary contraction. To the best of our knowledge, this is the first attempt at obtaining reference knee strength values by performing an all-encompassing review of the literature. Several previous studies have attempted to obtain reference values for specific populations or subgroups by performing measurements on larger samples of participants. Zvijac et al. (2014) examined over 1,200 collegiate male American football players and reported the means for peak isokinetic (60°/s) knee torque that ranged (based on the playing position and limb dominance) from 2.48 to 3.08Nm/kg and from 1.69 to 2.10Nm/kg for extension and flexion, respectively. Furthermore, Whiteley et al. (2012) examined 216 professional male soccer players and reported mean isokinetic (60°/s) knee extension strength at 3.1–3.3Nm/kg and knee flexion strength at 1.7–1.8Nm/kg. Hannon et al. (2019), likewise using isokinetic assessment at 60°/s, assessed elite female handball (*n*=150) and soccer (*n*=200) players, and observed a similar normalized peak torque in both player groups (mean extension: 2.3–2.4Nm/kg; mean flexion: 1.3–1.4Nm/kg). Similar to our results, the extension torque was highest in the mid-range knee position, while the flexion torque was relatively constant across the range of motion. In sum, the values obtained in the previous studies roughly agree with our results. Somewhat lower torque values sometimes obtained in the abovementioned studies likely resulted from using isokinetic measurements. According to the force-velocity relationship (Perrine and Edgerton, 1978; Gülch, 1994; Grbic et al., 2017), isokinetic assessment should result in lower values than isometric measurements.

For the non-athletic populations, comparing our results to the available literature is challenging, because previous studies that attempted to obtain reference values did not report body-mass normalized units (Meldrum et al., 2007; Harbo et al., 2012; McKay et al., 2017). Interestingly, our analyses showed only modest differences between athletes and general adult population. Accordingly, Ushiyama et al. (2017) reported the mean isometric knee extension torque of 2.64Nm/kg (range: 1.98–4.61) in 30 (20 males) healthy adult participants. Some studies reported values exceeding the confidence intervals that we calculated. For instance, Dalgaard et al. (2019) reported isometric knee extension (knee angle=70°) in 28 young females at 3.0Nm/kg, and even at 3.3–3.5Nm/kg after a period of resistance training. Unfortunately, to the best of our knowledge, no study examined knee strength in a larger sample (e.g., >100) of adults and reported the outcomes in Nm/kg. Harbo et al. (2012) tested 93 male and 85 female non-athlete adults and reported isometric knee extension peak torque at 246.6±56.3Nm for the males and 166.6±38.2Nm for the females, which is within our calculated confidence intervals for non-normalized values (although close to the upper limit; Supplementary File 1).

The reference values that we calculated for the older adults also seem to mostly agree with individual previous studies. Periera et al. examined the isokinetic (60°/s) knee extension strength in 453 older women, separated by age groups. In the 60–65years old group, they reported the mean knee extension peak torque values at 1.5Nm/kg, which corresponds to our results for mid-range knee angle measurements. Interestingly, they observed only a modest decline in strength throughout the groups, reaching 1.2Nm/kg in the oldest group (80–85years old), with barely noticing any decline until 75years of age. This supports our decision to pool all available data from the older adult studies since most of these included older adults aged between 65 and 75 years. Spinoso et al. (2019) also assessed isometric knee flexion strength (knee angle=60°) in 30 older females and reported the mean value at 0.64±0.17Nm/kg, which was statistically significantly lower than the mean value in the group of 16 younger female participants (1.09±0.23Nm/kg). A similar observation was reported for the isometric knee extension strength (1.65±0.23Nm/kg in the older group; 2.45±0.52Nm/kg in the younger group; Spinoso et al., 2019). These results all fit the knee strength reference values that we calculated. Lower mean values for isometric knee extension strength (1.12–1.15Nm/kg) were reported by Kim et al. (2012) for 55 community-dwelling elderly women (aged 75 and older) with sarcopenia. Lower-limb muscle strength is one of the determinants of risk of falling (Pijnappels et al., 2008; Hicks et al., 2020) and is also associated with general physical functioning of older adults (Liu and Latham, 2009; Brady et al., 2014). Therefore, the values obtained in this review could serve as a reference when trying to improve strength in older adults.

Some limitations of this review need to be acknowledged and discussed. The authors generally followed the PRISMA guidelines, with some exceptions being made that are limiting the rigor of this review. Firstly, although the authors performed the review with the best possible care and scrutiny, the relevant body of literature was large, which makes it possible that some studies were omitted. Nevertheless, we believe that a significant proportion of the relevant studies have been included in the review, and additional studies would have no or small effects on the pooled results. For the same reason, only two literature databases were considered. The risk of bias of individual studies was not assessed. This was mainly due to the fact that a multitude of study designs were eligible for inclusion in the review, which make the comparison of papers very difficult. Moreover, we omitted some of the possible bias sources (e.g., allocation bias) by extraction only baseline data in longitudinal studies. However, other types of bias, such as selection bias, could have still affected our results. Unfortunately, several studies that examined knee strength did not report descriptive data. Normalization of the data to Nm/kg was also not possible in all studies because body mass data were absent. Furthermore, it is not entirely clear whether normalizing the torque values by body mass is always the most appropriate. The first studies that examined the methods used for adjusting strength values to body size suggested that torque values should indeed be divided by body mass, while force values should be divided with 2/3 of the body mass for appropriate between-participant comparisons (Jaric et al., 2002). However, some of the subsequent studies showed that different calculations might be more suitable in different populations. For instance, Abdalla et al. (2020) suggested that the knee extension strength should be divided by 96% of body mass in older males and 70% of body mass in older females when establishing sarcopenia status. For children, it was suggested that 140% of the body mass should be used in the division (Wren and Engsberg, 2007). This does not affect our results, which represent pooled data from multiple studies that reported body mass normalized values as torque divided by 100% of the body mass. Rather, future researchers should be careful when using our values to establish different criteria or cut-offs.

## Conclusion

This review provides reference values for isometric single-joint knee flexion and extension strength. The obtained values seem to mostly agree with previous single studies that attempted to obtain reference knee strength values in specific subgroups. These values may serve as a reference for future researchers, as well as practitioners during rehabilitation of musculoskeletal injuries, prevention or general assessment of physical abilities of the older adult or patient populations. Researchers and practitioners should be mindful of factors affecting knee extension and flexion strength (and the ratio of the two), such as sex, age, and knee angle. We have to stress that the values should be viewed cautiously, as some factors influencing strength could not be taken into account.

## Author Contributions

NS conceptualized the paper. NS and ZK carried out the study selection process. MP and ZK analyzed the data. ZK wrote the first manuscript draft. MP and NS finalized the paper. All authors contributed to the article and approved the submitted version.

## Funding

The study was supported by the Slovenian Research Agency through the project TELASI-PREVENT (L5-1845; Body asymmetries as a risk factor in musculoskeletal injury development: studying aetiological mechanisms and designing corrective interventions for primary and tertiary preventive care). The funder played no role in conceptualization of the study, data acquisition, article writing nor any other phase of the study.

## Conflict of Interest

NS was employed by company S2P, Science to Practice, Ltd.

The remaining authors declare that the research was conducted in the absence of any commercial or financial relationships that could be construed as a potential conflict of interest. Authors alone are responsible for conceptualization of the study and data analysis. The funders provided part of the authors’ salaries through national research fund and were not involved in any part of the study.

## Publisher’s Note

All claims expressed in this article are solely those of the authors and do not necessarily represent those of their affiliated organizations, or those of the publisher, the editors and the reviewers. Any product that may be evaluated in this article, or claim that may be made by its manufacturer, is not guaranteed or endorsed by the publisher.
