# Supplementary material for: Establishing Reference Values for Isometric Knee Extension and Flexion Strength
Source: Front Physiol. 2021 Oct 15;12:767941. doi: 10.3389/fphys.2021.767941 (PMC8554160; doi:10.3389/fphys.2021.767941)
Supplement: Supplementary file 1 [file Table_1.pdf]

Supplementary file 1 – Part A (Main analysis)

KNEE ANGLE	Grouup	Gender	Task	Body-mass normalized (Nm/kg)					Non-normalized (Nm)				
				MEAN	95 % CI		Subjects	Studies	MEAN	95 % CI		Subjects	Studies
Extended	Athletes	MALE	Extension	1.872	1.584	2.160	543	25	137.9	121.3	154.5	282	20
Extended	Athletes	MALE	Flexion	1.535	1.182	1.888	292	4	169.7	107.9	231.4	47	3
Extended	Adults	BOTH	Extension	1.613	1.293	1.934	423	18	116.0	98.3	133.8	397	17
Extended	Adults	MALE	Extension	1.788	1.345	2.232	286	10	139.5	97.4	181.7	223	6
Extended	Adults	FEMALE	Extension	1.258	1.013	1.502	95	5	82.0	69.4	94.6	105	6
Extended	Adults	BOTH	Flexion	1.052	0.916	1.188	183	11	81.4	74.1	88.8	201	12
Extended	Adults	MALE	Flexion	1.025	0.853	1.197	99	5	84.5	69.3	99.8	117	6
Extended	Adults	FEMALE	Flexion	0.996	0.779	1.212	82	5	68.7	65.5	71.9	68	4
Extended	Elderly	BOTH	Extension	1.041	0.842	1.240	213	15	70.1	51.8	88.4	220	11
Extended	Elderly	MALE	Extension	1.158	0.948	1.367	132	9	64.1	52.0	76.3	42	3
Extended	Elderly	FEMALE	Extension	0.624	0.180	1.068	40	3	42.7	38.3	47.0	40	3
Extended	Elderly	BOTH	Flexion	0.860	0.651	1.068	112	9	64.0	51.9	76.0	209	11
Extended	Elderly	MALE	Flexion	0.895	0.592	1.197	42	3	74.6	67.1	82.2	42	3
Extended	Elderly	FEMALE	Flexion	0.579	0.057	1.102	60	5	59.4	42.4	76.4	70	6
Mid-range	Athletes	BOTH	Extension	3.110	2.879	3.341	806	51	237.5	224.3	250.8	729	43
Mid-range	Athletes	MALE	Extension	3.163	2.897	3.429	455	34	251.4	234.0	268.7	404	31
Mid-range	Athletes	FEMALE	Extension	3.092	2.877	3.286	81	5	199.8	170.4	229.1	20	2
Mid-range	Athletes	BOTH	Flexion	1.530	1.396	1.664	258	13	134.2	110.9	157.5	258	11
Mid-range	Athletes	MALE	Flexion	1.637	1.364	1.910	105	7	154.5	137.1	172.0	142	7
Mid-range	Athletes	FEMALE	Flexion	1.554	1.497	1.611	61	3	/	/	/	/	/
Mid-range	Adults	BOTH	Extension	2.680	2.390	2.970	1839	44	191.0	176.3	205.8	1928	46
Mid-range	Adults	MALE	Extension	3.187	2.921	3.452	937	15	234.5	204.1	264.8	940	15
Mid-range	Adults	FEMALE	Extension	2.377	2.018	2.736	707	15	150.8	133.1	168.5	701	13
Mid-range	Adults	BOTH	Flexion	1.233	1.026	1.440	933	13	82.7	65.6	99.9	990	13

Mid-range	Adults	MALE	Flexion	1.387	1.153	1.622	832	7	105.3	97.0	113.7	827	6
Mid-range	Adults	FEMALE	Flexion	0.937	0.817	1.058	66	4	55.8	50.3	61.3	128	5
Mid-range	Elderly	BOTH	Extension	1.682	1.561	1.803	1781	41	121.5	111.9	131.2	1677	38
Mid-range	Elderly	MALE	Extension	1.950	1.743	2.158	534	14	163.9	146.6	181.3	400	10
Mid-range	Elderly	FEMALE	Extension	1.519	1.401	1.638	1156	19	102.3	95.0	109.7	1000	20
Mid-range	Elderly	BOTH	Flexion	0.705	0.611	0.798	286	11	50.9	44.5	57.3	377	13
Mid-range	Elderly	MALE	Flexion	0.791	0.688	0.894	58	4	66.7	61.8	71.7	58	4
Mid-range	Elderly	FEMALE	Flexion	0.635	0.462	0.808	78	6	43.6	37.5	49.7	169	8
Flexed	Athletes	BOTH	Extension	2.510	2.179	2.841	663	43	200.5	186.7	214.3	580	36
Flexed	Athletes	MALE	Extension	2.721	2.335	3.108	342	23	190.9	177.6	204.2	368	25
Flexed	Athletes	FEMALE	Extension	1.167	1.055	1.278	92	3	/	/	/	/	/
Flexed	Athletes	BOTH	Flexion	1.134	0.683	1.586	99	7	/	/	/	/	/
Flexed	Athletes	MALE	Flexion	1.469	0.950	1.988	42	4	145.6	108.8	182.4	72	4
Flexed	Athletes	FEMALE	Flexion	/	/	/	/	/	/	/	/	/	/
Flexed	Adults	BOTH	Extension	2.657	2.483	2.830	4905	71	200.2	188.7	211.7	4884	71
Flexed	Adults	MALE	Extension	2.776	2.496	3.057	3718	27	225.2	208.1	242.2	3858	35
Flexed	Adults	FEMALE	Extension	2.371	2.036	2.706	621	10	143.8	126.7	161.0	665	14
Flexed	Adults	BOTH	Flexion	1.185	1.034	1.335	505	23	88.7	66.7	110.8	389	17
Flexed	Adults	MALE	Flexion	1.253	0.963	1.543	267	12	92.7	59.3	126.0	217	9
Flexed	Adults	FEMALE	Flexion	1.075	0.459	1.691	32	2	68.3	38.6	97.9	32	2
Flexed	Elderly	BOTH	Extension	1.707	1.499	1.915	738	31	123.9	110.2	137.5	725	31
Flexed	Elderly	MALE	Extension	1.769	1.495	2.043	392	15	150.5	132.4	168.6	318	10
Flexed	Elderly	FEMALE	Extension	1.333	1.045	1.621	214	11	94.0	79.9	108.1	210	12
Flexed	Elderly	BOTH	Flexion	0.636	0.423	0.848	149	10	55.7	43.5	68.0	135	10
Flexed	Elderly	MALE	Flexion	0.699	0.572	0.827	42	3	56.7	50.4	63.0	42	3
Flexed	Elderly	FEMALE	Flexion	0.451	0.338	0.563	60	4	32.6	25.8	39.3	60	4

Supplementary file 1 – Part B (Preliminary analysis)

Knee angle	Group	Gender	Task	Body-mass normalized (Nm/kg)					Non-normalized (Nm)				
				MEAN	95 % CI		Subjects	Studies	MEAN	95 % CI		Subjects	Studies
0-15	Sport (P)	BOTH	Extension	1.154	0.910	1.397	65	6	103.4	94.4	112.4	56	5
20	Elderly	BOTH	Extension	1.226	1.092	1.360	92	6	50.3	47.8	52.8	166	7
20	Elderly	BOTH	Flexion	0.693	0.255	1.131	102	8	52.1	49.5	54.6	186	9
20	Sport (P)	BOTH	Extension	1.504	1.265	1.743	65	6	142.6	139.9	145.2	56	5
30	Adults	BOTH	Extension	1.567	1.077	2.058	191	10	120.3	102.8	137.8	165	10
30	Adults	BOTH	Flexion	0.849	0.758	0.940	98	6	76.4	68.3	84.5	98	3
45	Sport (P)	BOTH	Extension	2.270	1.736	2.804	132	10	178.1	160.8	195.3	111	7
45	Sport (P)	BOTH	Flexion	1.303	1.076	1.530	12	2	/	/	/	/	/
45	Sport (P&R)	BOTH	Extension	2.402	2.271	2.533	137	5	192.2	158.7	225.8	87	4
45	Sport(P&R)	BOTH	Extension	2.322	1.932	2.712	269	15	183.1	168.5	197.7	198	11
45	Sport(P&R)	BOTH	Flexion	1.365	1.274	1.455	82	4	/	/	/	/	/
45	Elderly	BOTH	Extension	1.602	1.441	1.762	64	5	92.5	56.4	128.6	47	4
45	Elderly	BOTH	Flexion	/	/	/	/	/	89.1	76.0	102.2	23	2
45	Adults	BOTH	Extension	1.745	1.643	1.847	234	8	149.2	104.0	194.4	204	7
45	Adults	BOTH	Flexion	1.460	1.347	1.574	78	4	77.2	57.0	97.4	38	3
60	Sport (P)	BOTH	Extension	2.694	1.951	3.436	87	7	220.9	181.1	260.7	56	5
60	Sport (P)	BOTH	Flexion	1.420	1.092	1.748	31	2	/	/	/	/	/
60	Sport (R)	BOTH	Extension	2.743	2.122	3.365	304	15	237.1	212.7	261.4	308	15
60	Sport (P&R)	BOTH	Flexion	1.296	0.490	2.103	104	3	98.8	36.8	160.7	192	7
60	Sport (P&R)	BOTH	Extension	2.756	2.241	3.270	391	22	232.8	212.9	252.8	364	20
60	Sport (P&R)	BOTH	Flexion	1.482	1.334	1.630	135	6	125.4	86.8	164.0	164	
60	Adults	BOTH	Extension	2.386	2.157	2.615	1678	31	186.3	168.6	204.1	1733	33

60	Adults	BOTH	Flexion	1.416	1.334	1.498	976	14	87.9	71.3	104.4	993	13
60	Elderly	BOTH	Extension	1.624	1.366	1.881	1605	30	113.4	104.1	122.8	1542	31
60	Elderly	BOTH	Flexion	0.670	0.399	0.941	288	11	51.4	44.8	58.0	367	13
70	Adults	BOTH	Extension	3.058	2.850	3.266	377	22	209.2	187.9	230.6	391	21
70	Adults	BOTH	Flexion	0.895	0.361	1.428	64	3	68.2	46.6	89.9	64	3
70	Elderly	BOTH	Extension	1.529	1.203	1.855	978	12	131.6	76.5	186.8	917	8
70	Sport (P)	BOTH	Extension	2.898	2.320	3.476	183	14	225.5	193.1	258.0	136	9
70	Sport (P)	BOTH	Flexion	1.595	1.334	1.856	98	6	152.6	145.4	159.8	69	3
70	Sport (P&R)	BOTH	Extension	2.696	2.523	2.870	217	13	265.4	242.5	288.4	177	13
70	Sport (P&R)	BOTH	Extension	2.839	2.550	3.348	400	27	248.7	226.6	270.8	313	21
80	Adults	BOTH	Extension	2.668	1.550	3.787	119	7	236.1	187.1	285.1	119	7
80	Adults	BOTH	Flexion	1.214	0.085	2.512	38	3	111.4	71.0	151.8	43	3
80	Elderly	BOTH	Extension	1.711	1.476	1.947	79	5	149.4	130.5	168.4	65	4
80	Sport (R)	BOTH	Extension	2.612	2.373	2.852	214	11	215.6	165.0	266.1	118	7
80	Sport (R)	BOTH	Flexion	1.622	0.930	2.314	50	2	/	/	/	/	/
90	Elderly	BOTH	Extension	1.639	1.317	1.961	599	25	122.8	107.3	138.4	638	27
90	Elderly	BOTH	Flexion	0.583	0.261	0.905	169	11	54.7	43.4	66.1	146	10
90	Adults	BOTH	Extension	2.549	2.267	2.830	4341	47	203.4	188.9	217.9	4309	48
90	Adults	BOTH	Flexion	1.181	0.898	1.463	282	11	101.0	69.9	132.1	243	10
90	Sport (P)	BOTH	Extension	3.683	3.296	4.069	99	7	294.6	243.7	345.5	93	6
90	Sport (P)	BOTH	Flexion	1.333	0.857	1.808	49	3	128.3	99.6	156.9	85	4
90	Sport (R)	BOTH	Extension	2.627	2.174	3.080	291	13	243.5	218.0	269.0	228	11
90	Sport (P&R)	BOTH	Flexion	1.021	0.597	1.445	106	4	/	/	/	/	/
90	Sport (P&R)	BOTH	Extension	3.045	2.520	3.569	390	20	262.7	239.3	286.1	321	17
90	Sport (P&R)	BOTH	Flexion	1.150	0.821	1.478	155	7	130.9	106.2	155.6	105	5
90	Children	BOTH	Extension	2.296	1.697	2.895	310	9	102.2	80.4	124.1	220	5
90	Children	BOTH	Flexion	1.201	0.901	1.500	59	3	/	/	/	/	/
100	Sport (P)	BOTH	Extension	1.465	1.288	1.641	86	8	166.3	145.8	186.8	87	9
100	Adults	BOTH	Extension	1.727	1.435	2.018	1292	13	170.2	144.7	195.8	1270	15

100	Adults	BOTH	Flexion	0.821	0.115	1.528	68	3	63.5	53.0	74.0	68	3
60	Adults	MALES	Extension	3.019	2.464	3.573	812	8	223.2	181.2	265.2	815	8
60	Adults	FEMALES	Extension	2.216	1.811	2.622	621	9	147.6	127.2	167.9	621	9
60	Adults	MALES	Flexion	1.501	1.359	1.643	802	6	108.8	100.1	117.6	797	5
60	Adults	FEMALES	Flexion	0.952	0.560	1.345	32	2	55.0	47.6	62.4	94	3
60	Elderly	MALES	Extension	2.095	1.792	2.399	451	9	152.8	137.3	168.2	356	8
60	Elderly	FEMALES	Extension	1.344	1.291	1.397	1053	16	93.8	87.6	100.0	875	15
60	Elderly	MALES	Flexion	0.834	0.208	1.460	58	4	66.7	61.8	71.7	58	4
60	Elderly	FEMALES	Flexion	0.545	0.139	0.950	68	5	39.0	33.5	44.5	137	6
60	Sport (P&R)	MALES	Extension	3.372	2.929	3.814	170	12	261.4	234.9	288.0	164	11
60	Sport (P&R)	FEMALES	Extension	3.066	2.958	3.175	60	4	/	/	/	/	/
60	Sport (P&R)	MALES	Flexion	1.369	0.963	1.776	18	2	157.1	129.9	184.2	94.00	
60	Sport (P&R)	FEMALES	Flexion	1.548	1.488	1.608	50	2	/	/	/	/	/
70	Adults	MALES	Extension	3.471	3.025	3.917	110	6	250.9	232.9	268.8	110	6
70	Adults	FEMALES	Extension	2.713	2.214	3.212	97	5	157.8	127.1	188.6	83	4
70	Adults	MALES	Flexion	/	/	/	/	/	/	/	/	/	/
70	Adults	FEMALES	Flexion	0.870	0.301	1.439	34	2	57.9	44.3	71.4	34	2
70	Sport (P&R)	MALES	Extension	3.240	2.868	3.613	280	21	253.7	227.5	279.9	244	17
70	Sport (P&R)	FEMALES	Extension	3.266	2.360	4.173	21	3	/	/	/	/	/
70	Sport (P&R)	MALES	Flexion	1.711	1.380	2.042	87	5	152.6	145.4	159.8	69	3
90	Adults	MALES	Extension	2.582	2.121	3.043	3422	17	233.4	211.4	255.4	3438	19
90	Adults	FEMALES	Extension	2.152	1.714	2.590	587	10	151.4	132.7	170.2	631	13
90	Adults	MALES	Flexion	1.458	1.151	1.765	146	4	100.9	38.4	163.3	121	3
90	Elderly	MALES	Extension	2.050	1.804	2.296	307	9	147.8	128.3	167.3	307	9
90	Elderly	FEMALES	Extension	1.268	0.932	1.604	337	12	87.8	75.2	100.4	190	11
90	Elderly	MALES	Flexion	0.727	0.169	1.286	42	3	56.7	50.4	63.0	42	3
90	Elderly	FEMALES	Flexion	0.399	0.163	0.635	60	4	32.6	25.8	39.3	60	4
90	Sport	MALES	Extension	3.514	3.052	3.977	200	12	270.7	234.7	306.8	204	12

90	Sport	FEMALES	Extension	1.167	1.055	1.278	92	3	/	/	/	/	/
90	Sport	MALES	Flexion	1.462	1.100	1.825	69	3	130.9	106.2	155.6	105	5
90	Children	MALES	Extension	1.861	1.450	2.272	43	7	67.2	56.6	77.7	49	8
90	Children	FEMALES	Extension	1.374	1.019	1.730	37	4	74.9	59.4	90.3	41	6
90	Children	MALES	Flexion	1.116	0.780	1.452	45	6	38.9	32.4	45.5	49	8
90	Children	FEMALES	Flexion	1.095	0.871	1.319	6	3	39.9	37.8	42.0	8	4

P – professional; R – recreational; P&R – professional and recreational.
